# Supplementary material for: Molecular Recognition Patterns between Vitamin B12 and Proteins Explored through STD-NMR and In Silico Studies
Source: Foods. 2023 Jan 28;12(3):575. doi: 10.3390/foods12030575 (PMC9914923; doi:10.3390/foods12030575)
Supplement: Supplementary file 1 [file foods-12-00575-s001.zip › foods-2158414-supplementary.pdf]

Supplementary Materials

Molecular Recognition Patterns Between Vitamin B12 and different plant and animal Proteins Explored Through STD–NMR and Molecular docking

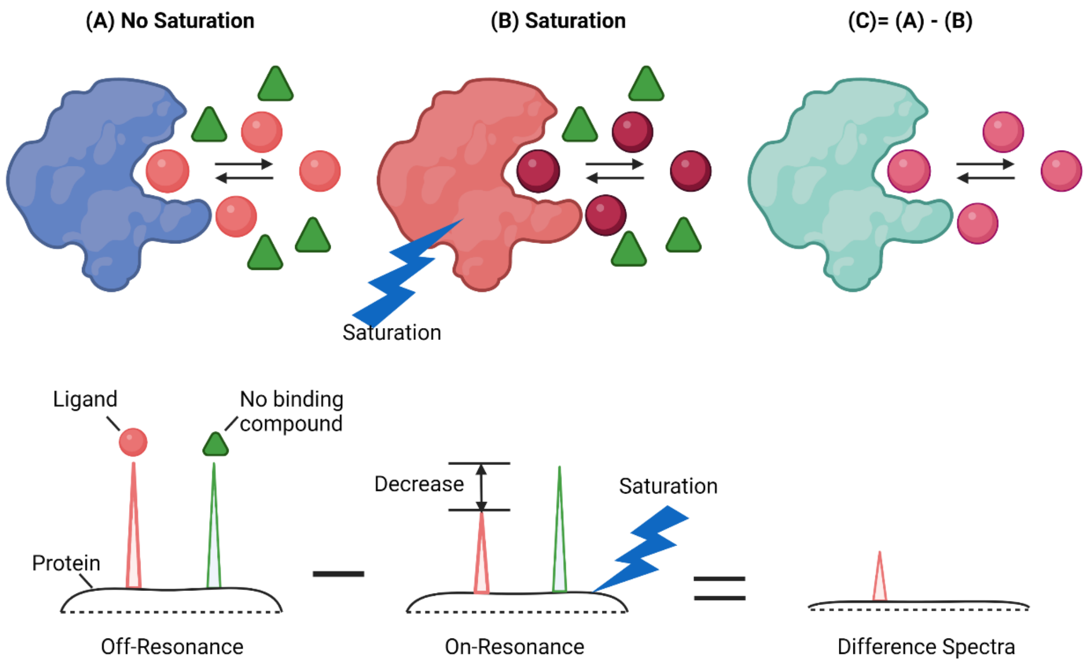

Fig. S1 STD-NMR experiment scheme. A typical STD-NMR spectrum was created bysubtracting spectra in which the protein signal was selectively saturated by irradiatingwith a low power pulse (on-resonance spectrum) from spectra in which the protein signal was not saturated (off-resonance spectrum)

Theoretical Concepts of STD–NMR

The nuclear Overhauser effect is the basis for STD-NMR (NOE). According to Fig. S1, when a radiofrequency pulse selectively saturates a subset of protein protons, spin diffusion across the protein results in the saturation being transferred from the protein to the ligand present at the protein's binding site, lowering the NMR intensity of ligand signals. To map the ligand's interaction epitope, keep in mind that the closer the ligand

comes into contact with the protein, the more saturation is produced and the lower the NMR intensity. To generate a typical STD-NMR spectra, the spectrum with the protein signal particularly saturated after being subjected to a low power pulse (on-resonance spectrum) was subtracted from the spectrum without protein saturation (off-resonance spectrum)[30]. A positive signal appears in this difference spectrum as a result of a transfer of magnetization from protein protons to the ligand, in which only the signals of the ligand engaging in the interaction remain. The degree of saturation will vary depending on which ligand protons are close to the protein. Then, a slow change from the ligand's free to bound states may send out a saturation, producing resonance signals with narrow line widths [31]. As an outcome, STD signals' strength reflects how close ligand protons are to the protein in space, which is helpful for locating binding epitopes. The STD strength (ISTD) is computed as[32] at a particular saturation time.

$$I_{STD} = \frac{I_0\%I_{sat}}{I_0} \quad \text{(S1)}$$

where I_{sat} is the signal intensity with selective saturation (on-resonance) and I_0 is the signal intensity without saturation (off-resonance). I_0–I_{sat} represents the intensity of the STD–NMR spectrum. The relative STD (%) signals were calculated by a normalization method to obtain a detailed identification of the groups involved in the binding with HSA. The strongest interacting proton was assigned an STD integral value of 100%, and the I_{STD} of other protons relative to the strongest STD effect was calculated:

$$\text{STD (\%)} = \frac{I_{STD(p*oton)}}{I_{STD(t_{-}e\ st*ongest\ p*oton)}} \quad \text{(S2)}$$

The STD amplification factor (A_{STD}), which is the relative intensity of the STD signal compared with that of a signal of the protein, was introduced to assess the absolute magnitude of the STD effect. It is a simple method for quantifying the amplification of the protein information observed in the STD signals of the ligand. A_{STD} is calculated by multiplying the ligand excess with this fractional STD effect according to the equation [33] :

$$A_{STD} = \frac{I_0\%I_{sat}}{I_0} \times \frac{[L]}{[P]} = I_{STD} \times \frac{[L]}{[P]} \quad \text{(S3)}$$

where *[L]* is the concentration of VB12, and *[P]* is the concentration of HSA. In its simplest form, ligand–protein binding follows a bimolecular association reaction. In the

case of fast exchange, A_{STD} at each ligand concentration was used to construct a binding (Langmuir) isotherm to obtain K_D with small bias. And a simple method based in the analysis of STD data that uses an analogy to the well-known Michaelis-Menten enzyme kinetics equation, can be written for the amplification factor to calculate K_D of HSA-VB12:

$$A_{STD} = \frac{\alpha_{STD} \times [L]}{K_D - [L]} \quad \text{(S4)}$$

where α_{STD} is a dimensionless scaling factor representing the maximum amplification factor. K_D value in STD–NMR experiments was obtained by a nonlinear least-squares-fitting curve using the Solver utility of Microsoft Excel. For comparison in this work, the dissociation constant (K_D) was converted into binding constant (Ka) in accordance with their reciprocal relation.

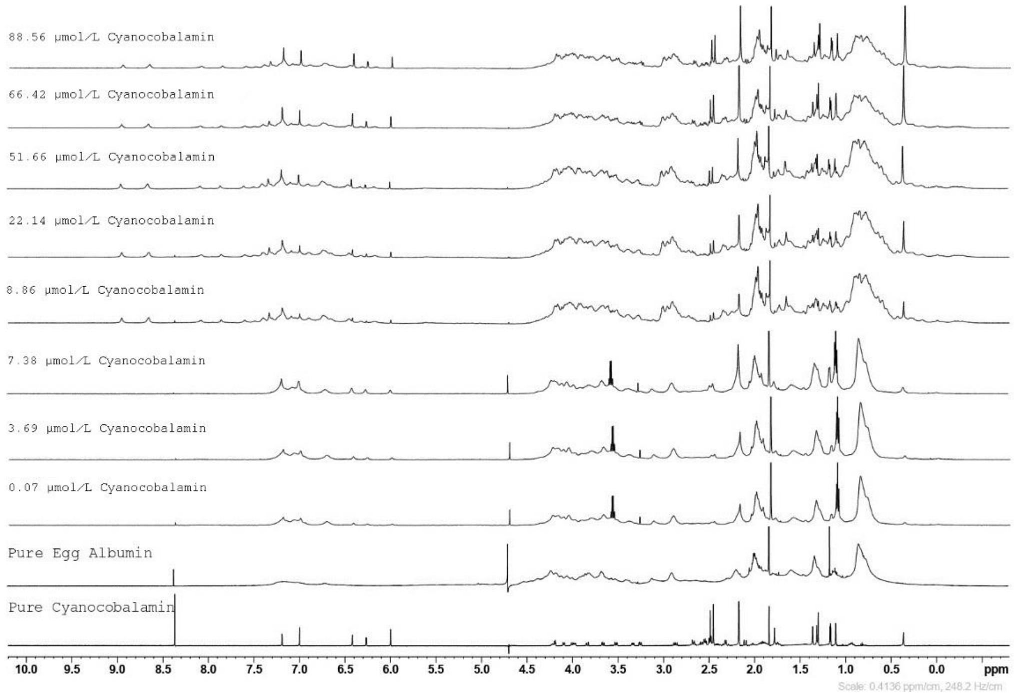

Fig. S2. STD–NMR Spectra of egg albumin and cyanocobalamin

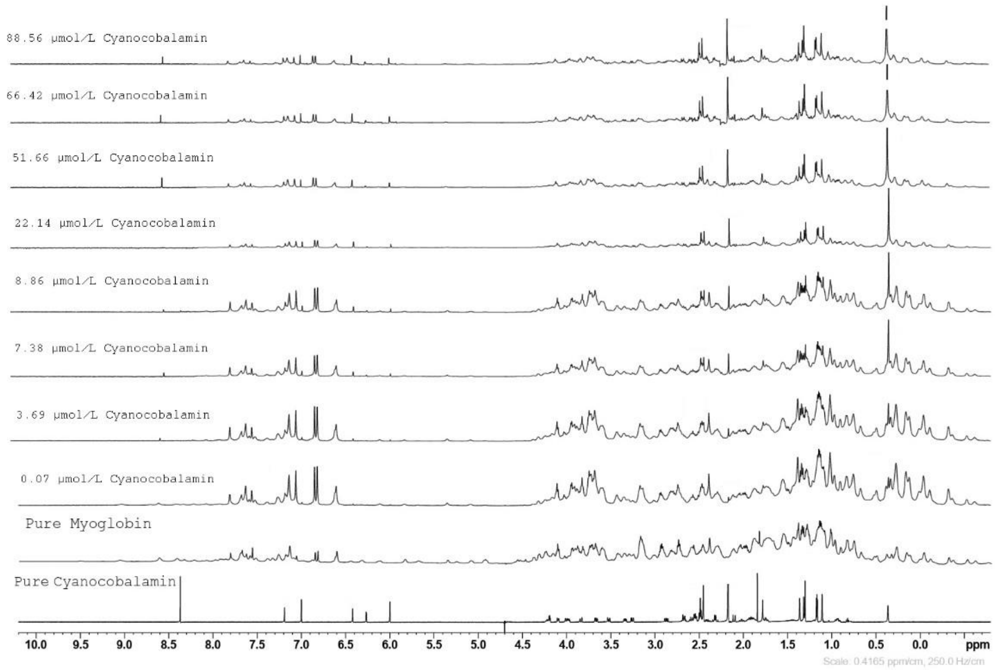

Fig. S3. STD–NMR Spectra of Myoglobin and cyanocobalamin

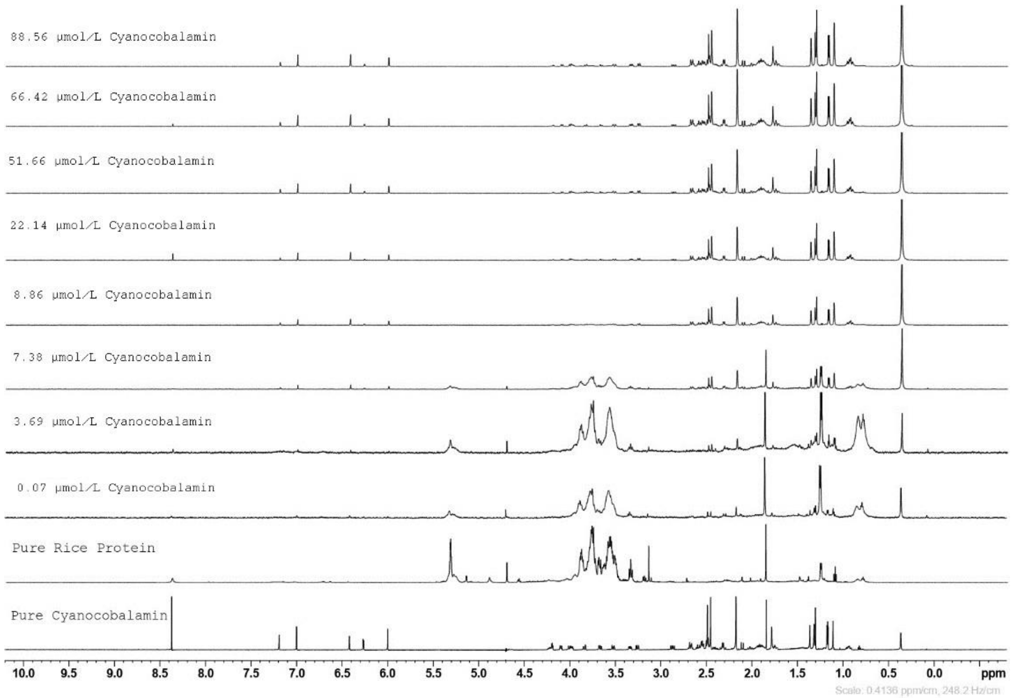

Fig. S4. STD–NMR Spectra of Rice protein and cyanocobalamin

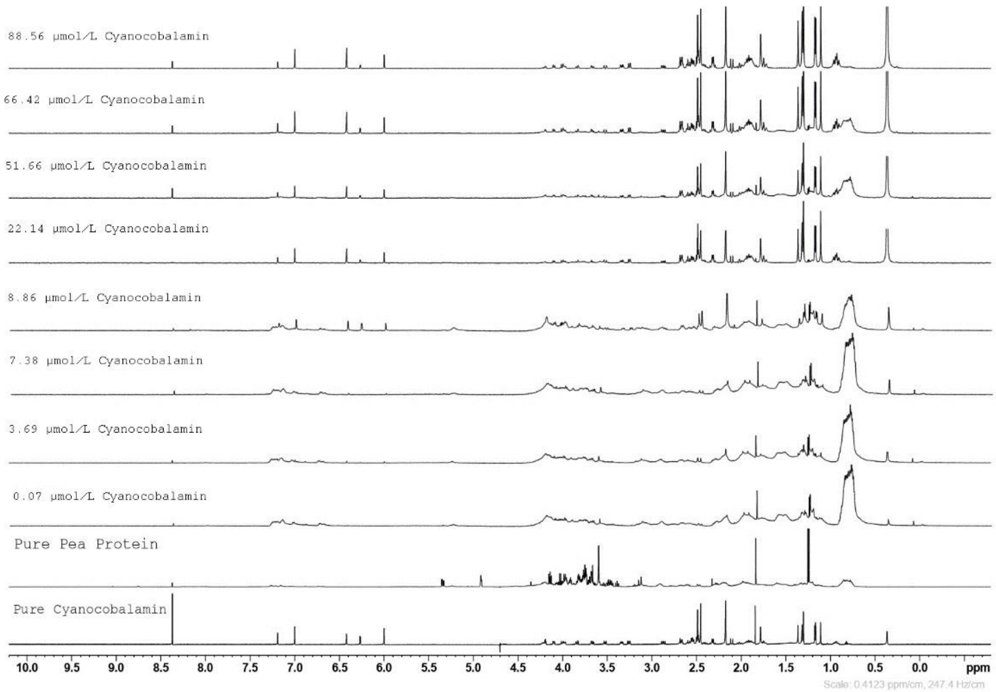

Fig. S5. STD–NMR Spectra of Pea Protein and cyanocobalamin

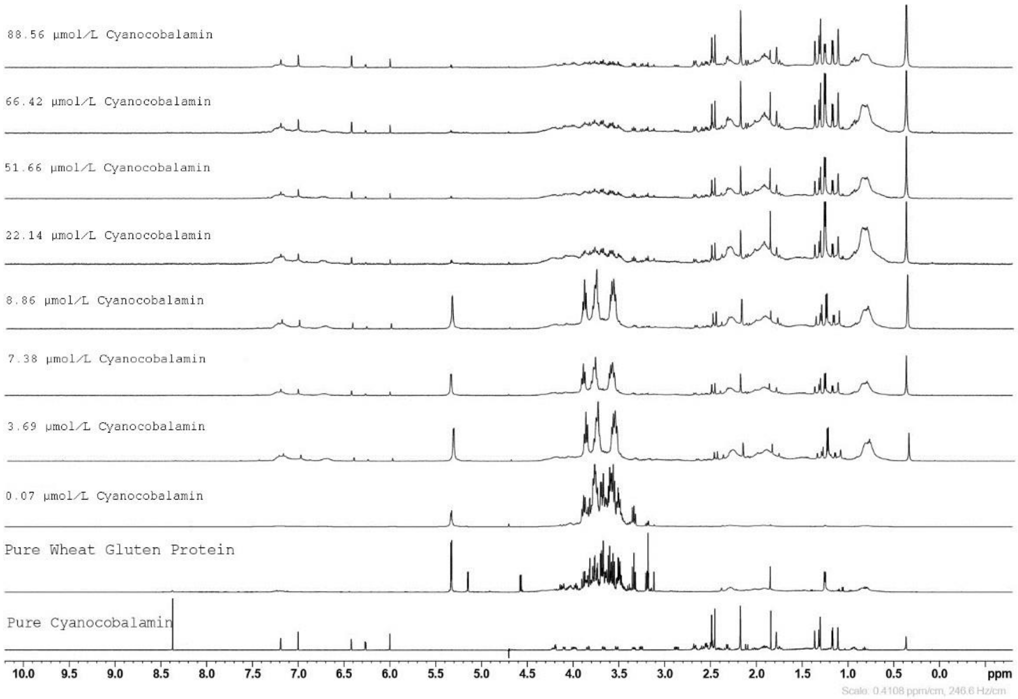

Fig. S6. STD–NMR Spectra of Gluten protein and cyanocobalamin

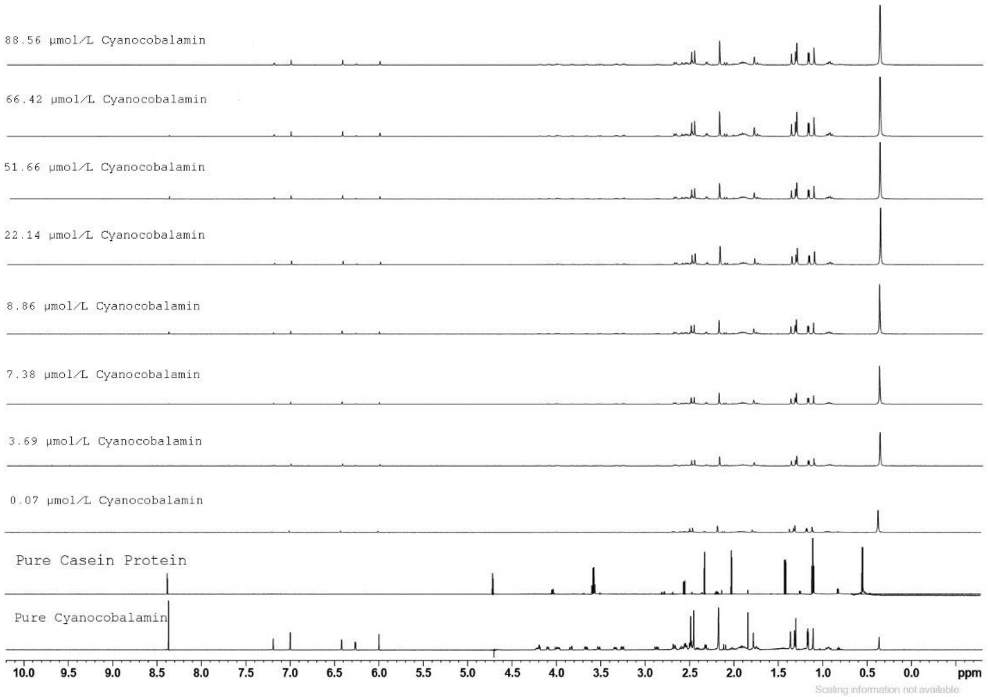

Fig. S7. STD–NMR Spectra of Casein and cyanocobalamin

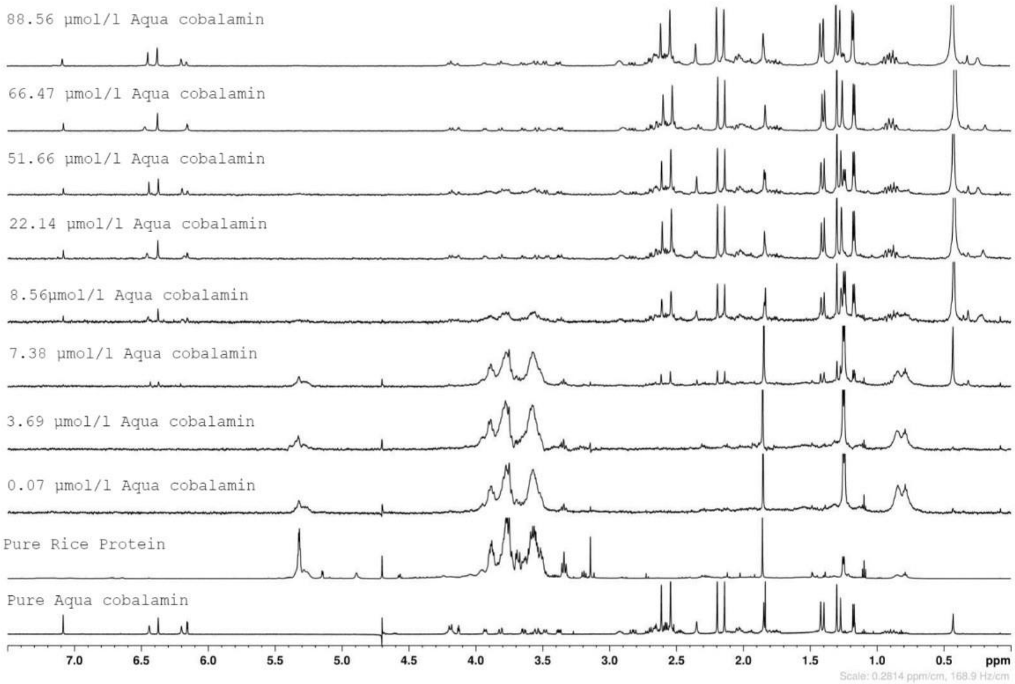

Fig. S8. STD–NMR Spectra of Rice protein and Aqua cobalamin

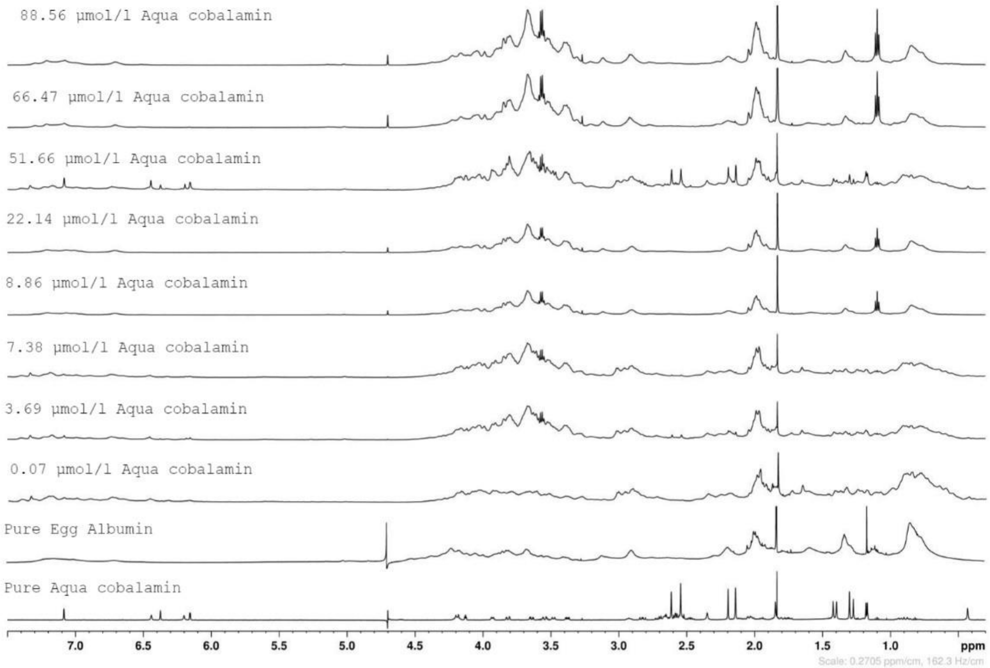

Fig. S9. STD–NMR Spectra of Egg Albumin and Aqua cobalamin

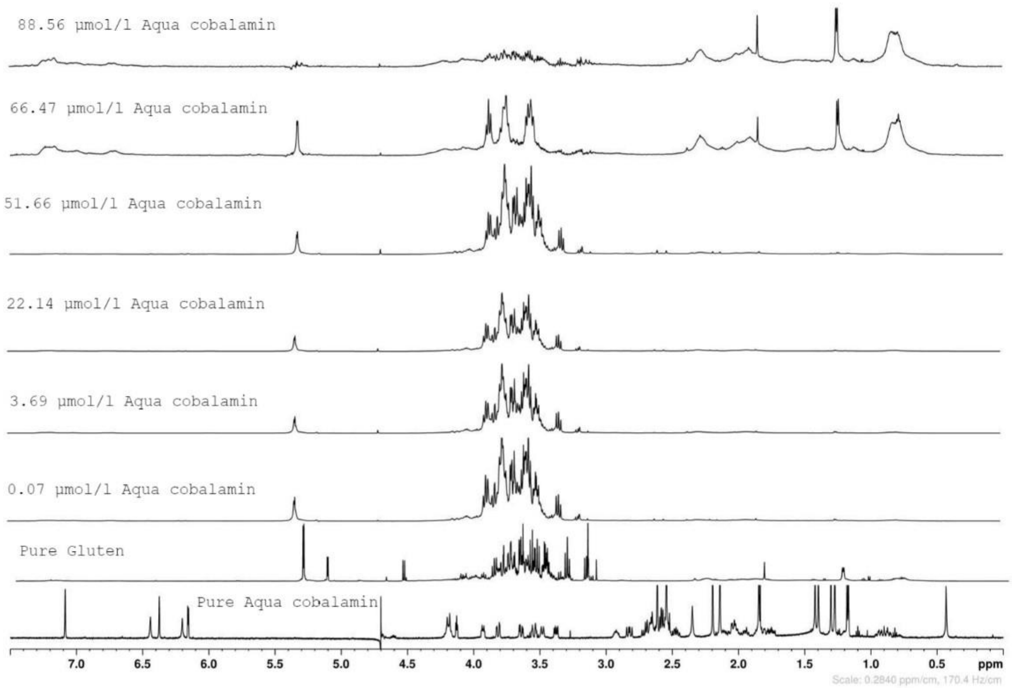

Fig. S10. STD–NMR Spectra of Gluten protein and Aqua cobalamin

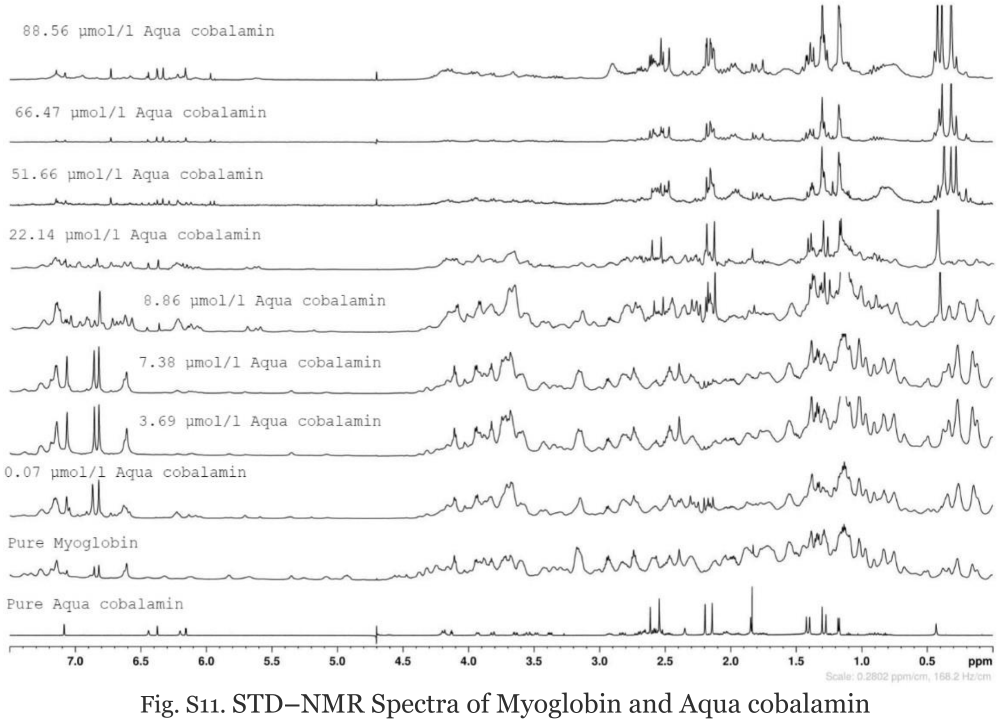

Fig. S11. STD–NMR Spectra of Myoglobin and Aqua cobalamin

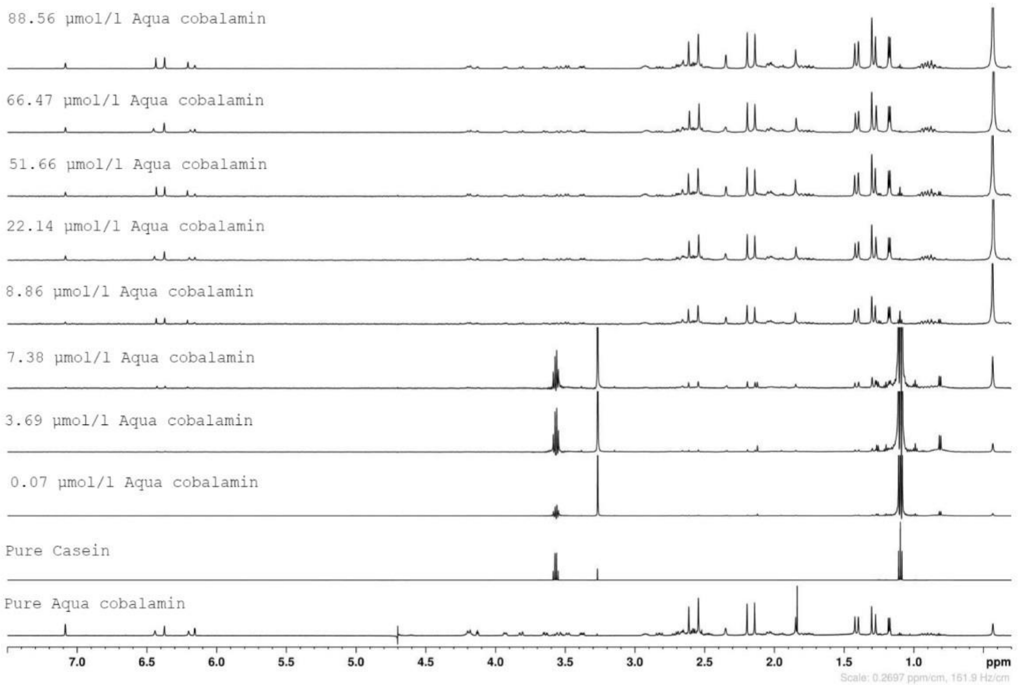

Fig. S12. STD−NMR Spectra of Casein and Aqua cobalamin

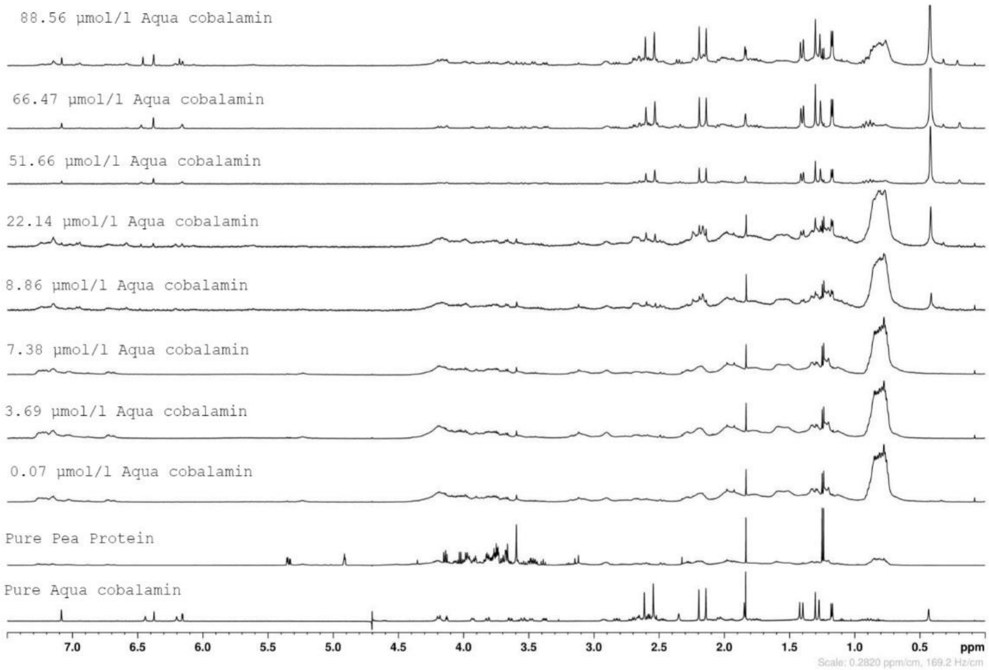

Fig. S13. STD−NMR Spectra of Pea protein and Aqua cobalamin

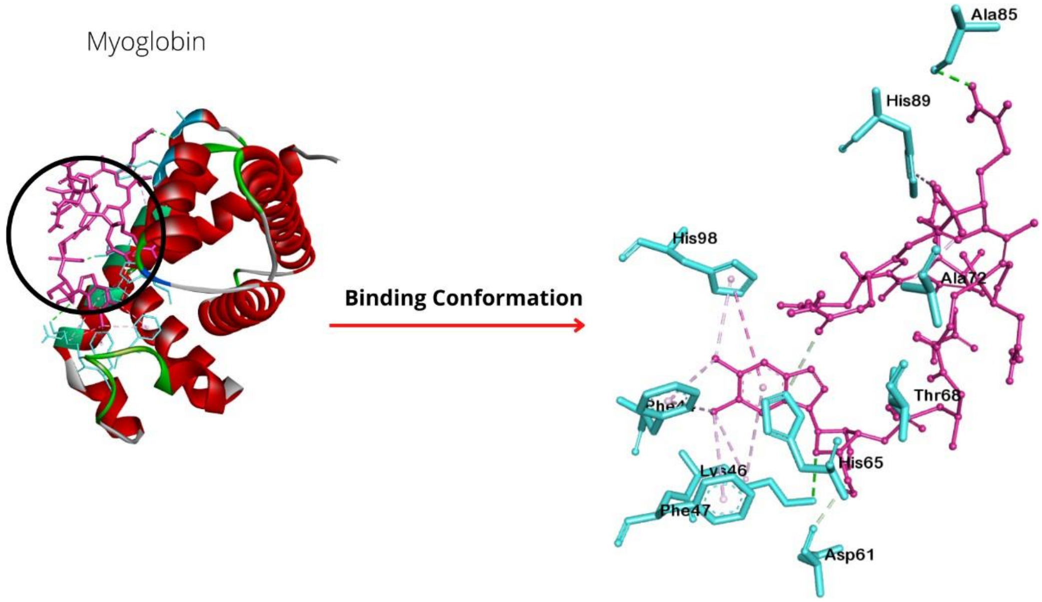

Fig. S14. Graphical representation of the binding conformation with the highest binding energy obtained from docking simulation. Myoglobin is colored light blue with labels of amino acid, VB12 colored red, hydrogen bonds colored green and π- bonds colored pink.

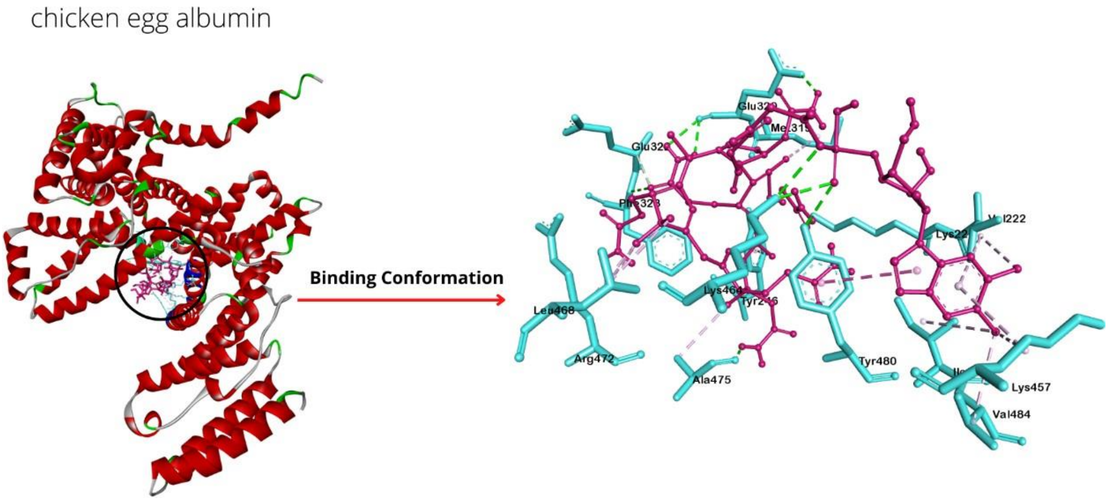

Fig. S15. Graphical representation of the binding conformation with the highest binding energy obtained from docking simulation. Chicken egg albumin is colored light blue with labels of amino acid, VB12 colored red, hydrogen bonds colored green and π- bonds colored pink.

casein

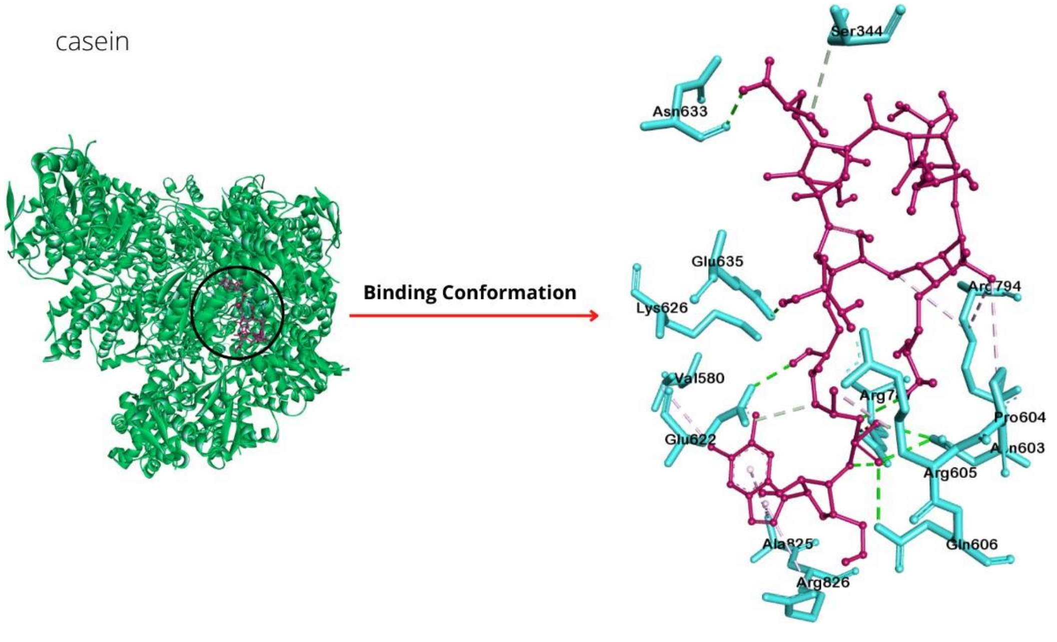

Fig. S16. Graphical representation of the binding conformation with the highest binding energy obtained from docking simulation. Casein colored light blue with labels of amino acid, VB12 colored red, hydrogen bonds colored green and π- bonds colored pink.

Rice

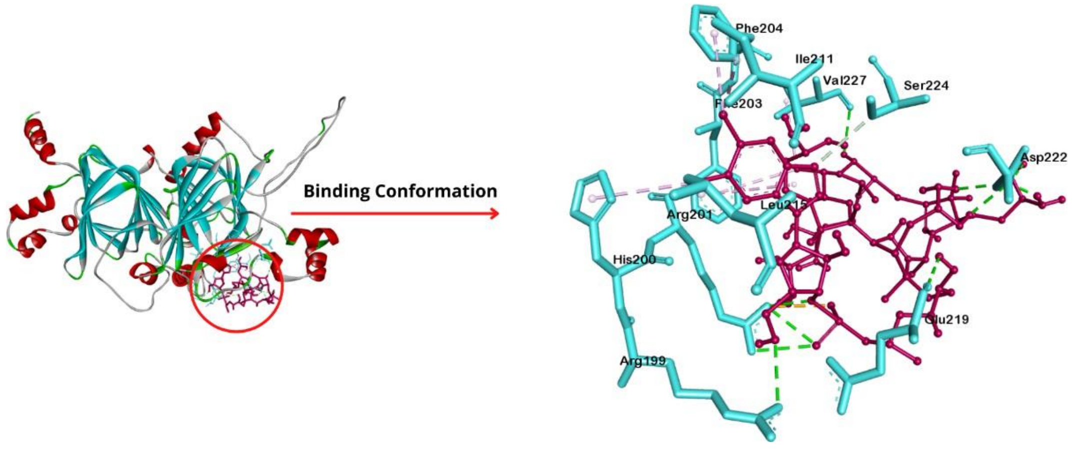

Fig. S17. Graphical representation of the binding conformation with the highest binding energy obtained from docking simulation. Rice protein Glutelin colored light blue with labels of amino acid, VB12 colored red, hydrogen bonds colored green and π- bonds colored pink.

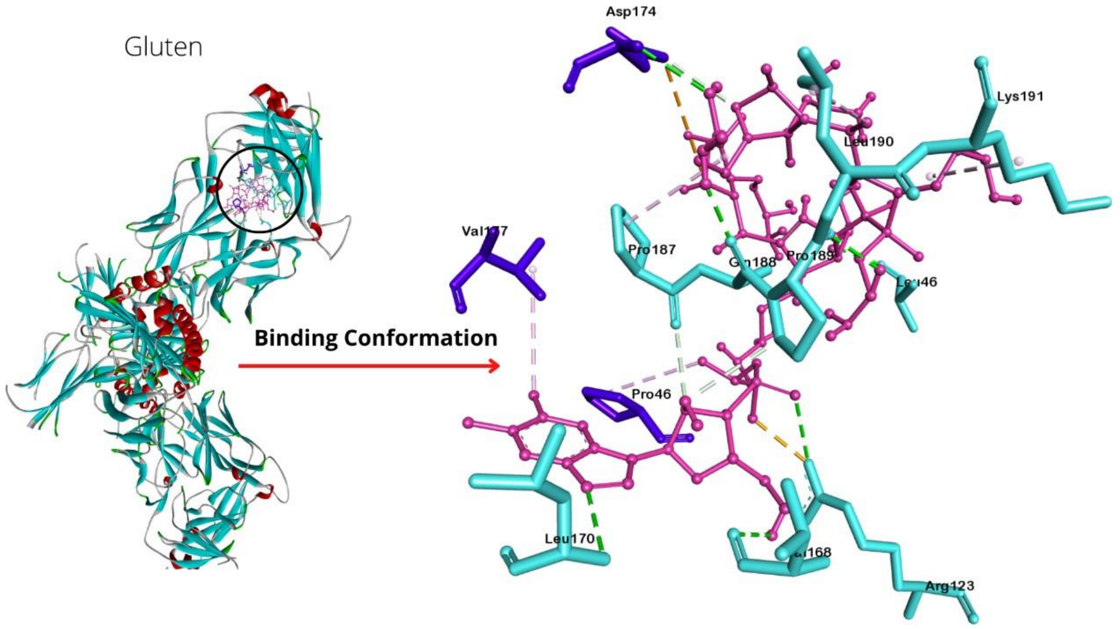

Fig. S18. Graphical representation of the binding conformation with the highest binding energy obtained from docking simulation. Gliadin from wheat Gluten is colored light blue(Chain G) and Blue (chain H) with labels of amino acid, VB12 colored red, hydrogen bonds colored green, and π- bonds colored pink.

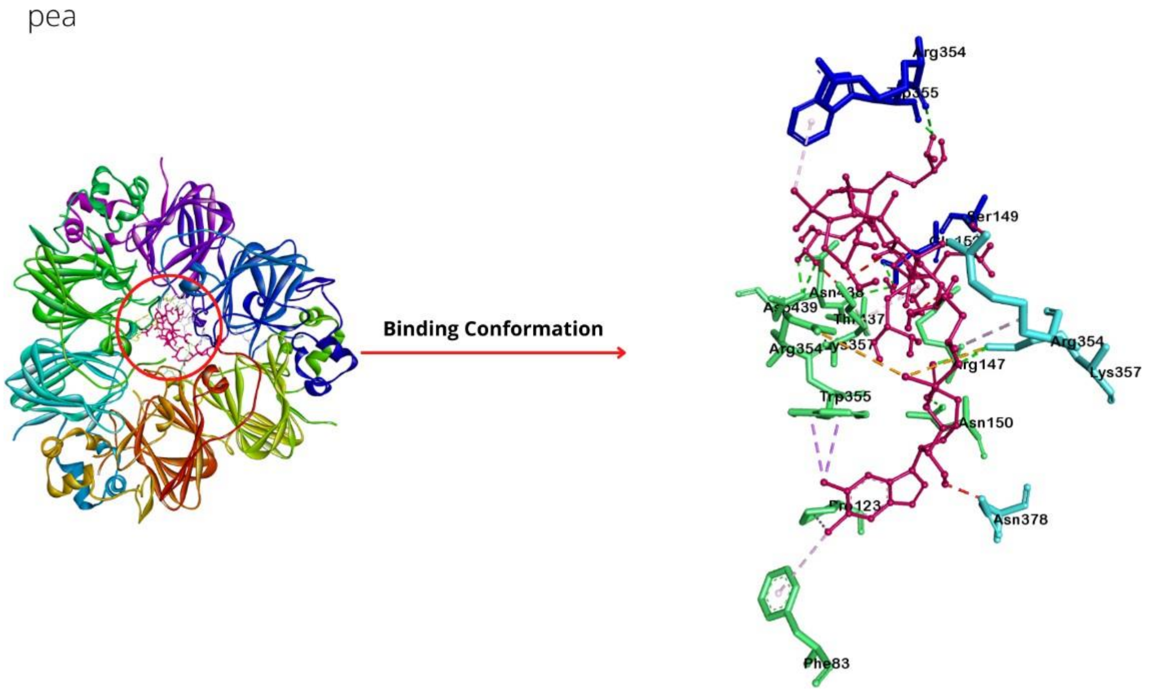

Fig. S19. Graphical representation of the binding conformation with the highest binding energy obtained from docking simulation. Pea protein colored light blue(Chain E), Green(Chain F), and Blue (chain G) with labels of amino acid, VB12 colored red, hydrogen bonds colored green, and π- bonds colored pink.

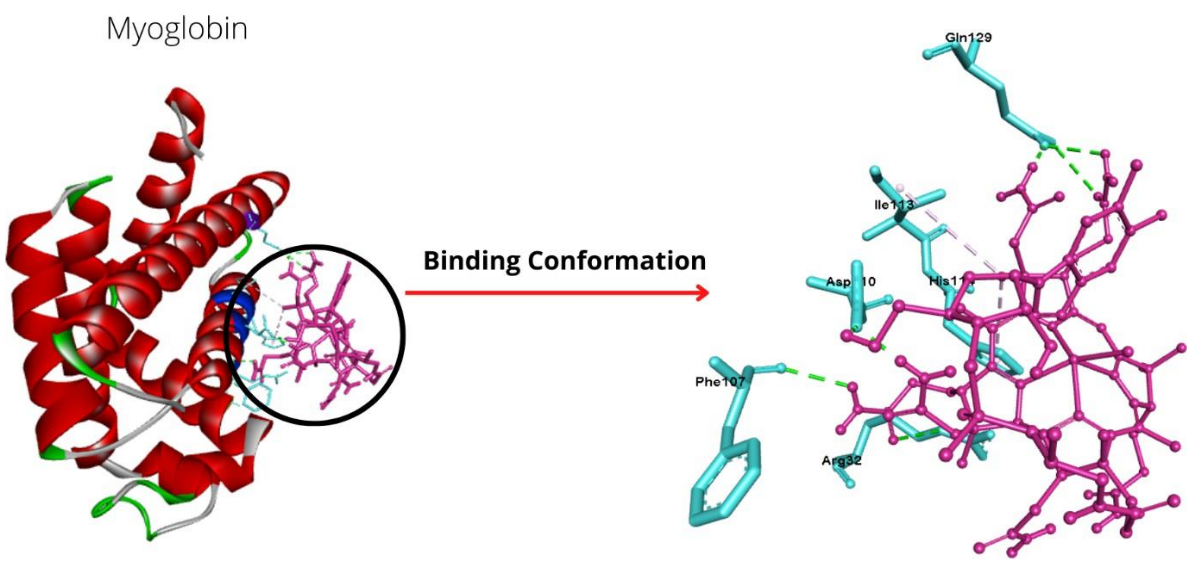

Fig. S20. Graphical representation of the binding conformation with the highest binding energy obtained from docking simulation. Myoglobin is colored light blue with labels of amino acid, hydroxy cobalamin colored red, hydrogen bonds colored green, and π- bonds colored pink.

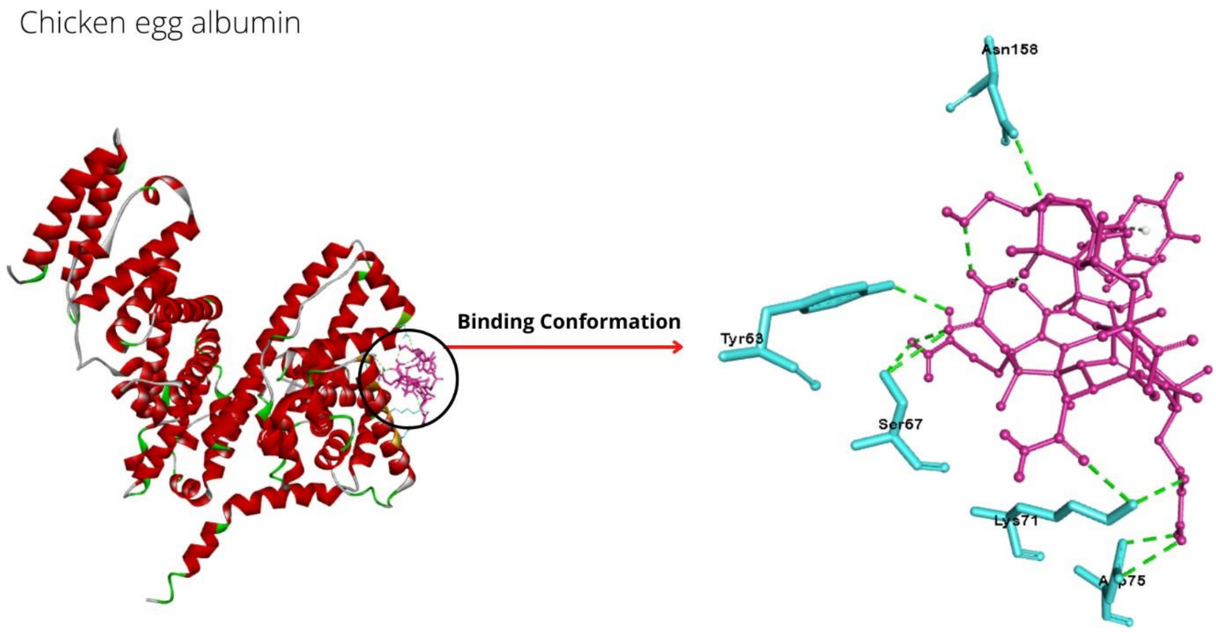

Fig. S21. Graphical representation of the binding conformation with the highest binding energy obtained from docking simulation. Chicken egg albumin is colored light blue with labels of amino acid, hydroxy cobalamin colored red, hydrogen bonds colored green, and π- bonds colored pink

Casein

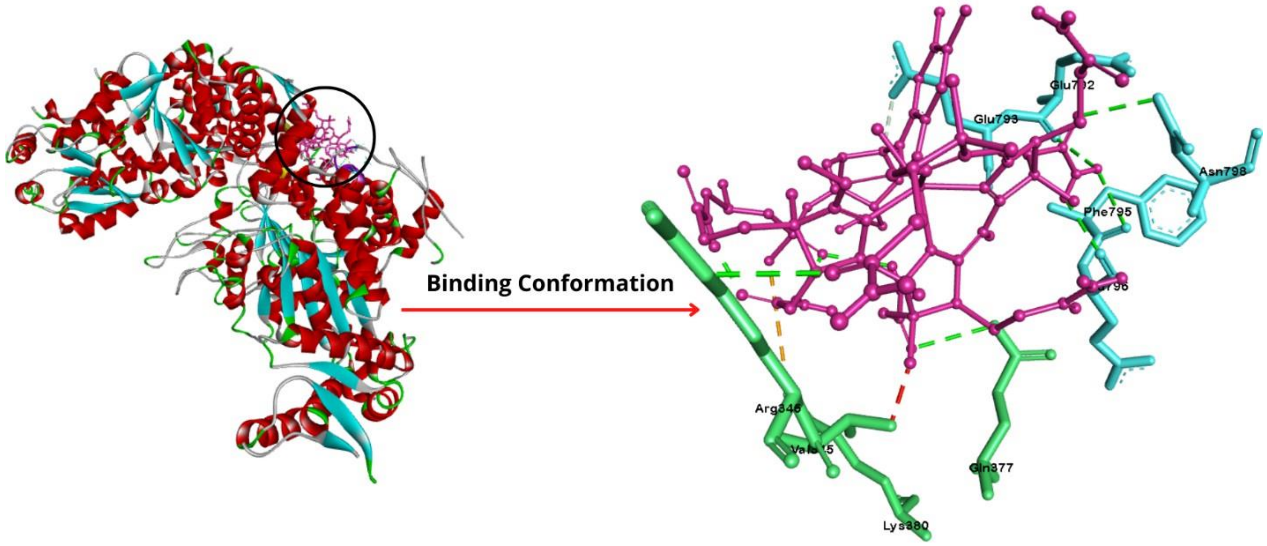

Fig. S22. Graphical representation of the binding conformation with the highest binding energy obtained from docking simulation. Casein colored light blue with labels of amino acid, hydroxy cobalamin colored red, hydrogen bonds colored green and π- bonds colored pink.

Rice

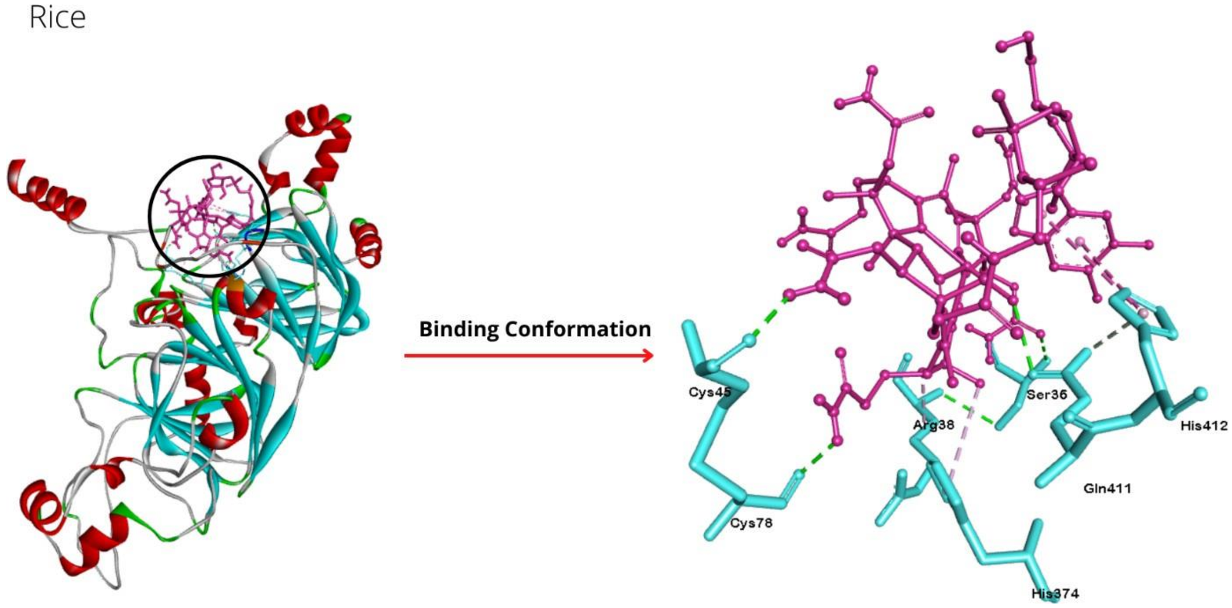

Fig. S23. Graphical representation of the binding conformation with the highest binding energy obtained from docking simulation. Rice protein Glutelin colored light blue with labels of amino acid, hydroxy cobalamin colored red, hydrogen bonds colored green andπ- bonds colored pink.

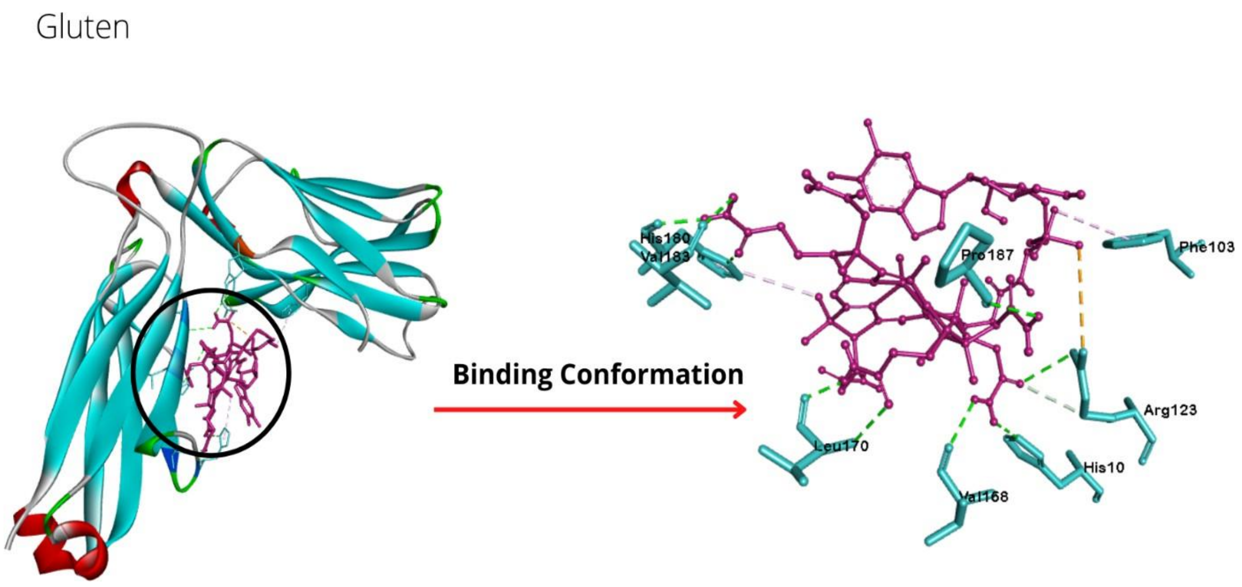

Fig. S24. Graphical representation of the binding conformation with the highest binding energy obtained from docking simulation. Gliadin from wheat Gluten is colored light blue with labels of amino acid, hydroxy cobalamin colored red, hydrogen bonds colored green, and π- bonds colored pink.

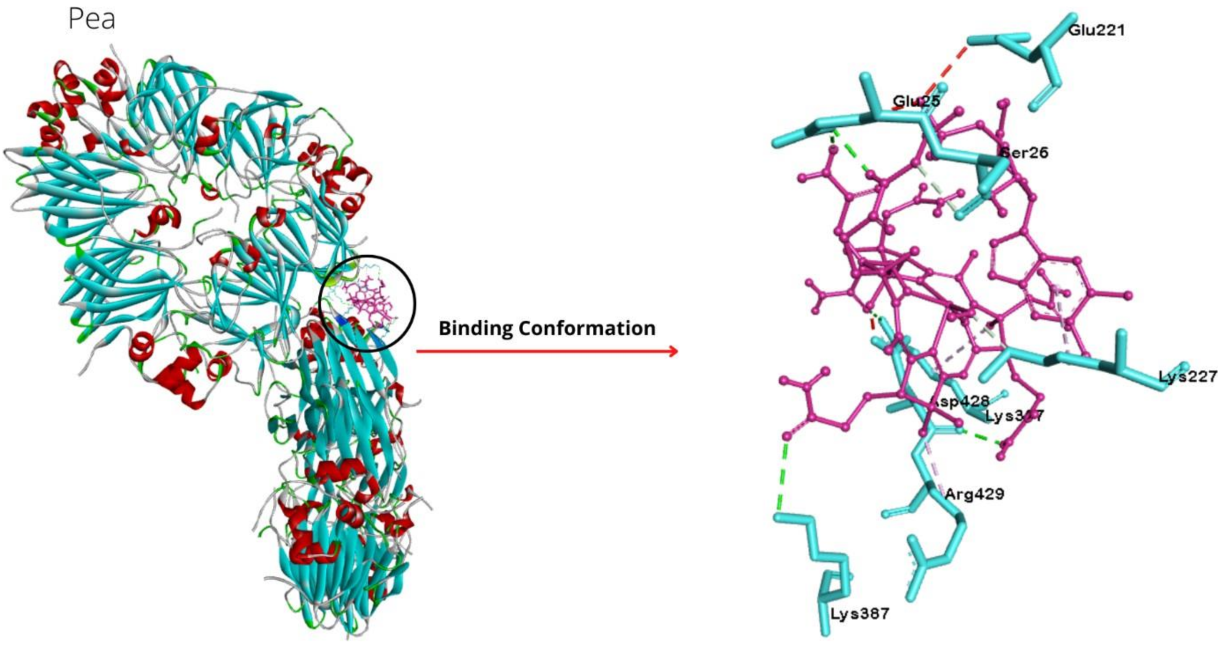

Fig. S25. Graphical representation of the binding conformation with the highest binding energy obtained from docking simulation. Pea protein is colored light blue with labels of amino acid, hydroxy cobalamin colored red, hydrogen bonds colored green, and π- bonds colored pink.
